# Presepsin to safely reduce antibiotics in preterm infants (PRESAFE): study protocol for a randomized controlled trial with a concurrent observational study

**DOI:** 10.1186/s13063-026-09515-8

**Published:** 2026-02-27

**Authors:** Charlotte M. Nusman, H. Rob Taal, Daniel C. Vijlbrief, Vincent Bekker, Karen W. G. van Mechelen, Willem P. de Boode, Marieke A. C. Hemels, Carmen M. Lorente Flores, Annemiek M. Roescher, Debbie H. G. M. Nuytemans, Sylvia A. Obermann-Borst, Michiel van der Flier, Leti van Bodegom-Vos, Christian R. B. Ramakers, Hans H. M. Schotman, Corianne A. J. M. de Borgie, Maruschka P. Merkus, Mariska M. G. Leeflang, Anton H. van Kaam, Wes Onland, Douwe H. Visser

**Affiliations:** 1 Department of Neonatology, Emma Children’s Hospital, Amsterdam University Medical Centres, Amsterdam, the Netherlands; 2 Amsterdam Reproduction & Development, Amsterdam, the Netherlands; 3 Department of Neonatal and Paediatric Intensive Care, Division of Neonatology, Erasmus University Medical Centre, Rotterdam, The Netherlands; 4 Department of Neonatology, Wilhelmina Children’s Hospital, University Medical Centre Utrecht, Utrecht, the Netherlands; 5 Department of Neonatology, Willem-Alexander Children’s Hospital, Leiden University Medical Centre, Leiden, the Netherlands; 6 Department of Neonatology, MosaKids Children’s Hospital, Maastricht University Medical Centre, Maastricht, the Netherlands; 7 Department of Neonatology, Amalia Children’s Hospital, Radboud University Medical Centre, Nijmegen, the Netherlands; 8 Department of Neonatology, Isala Women and Children’s Hospital, Zwolle, the Netherlands; 9 Department of Neonatology, Maxima Medical Centre, Veldhoven, the Netherlands; 10 Department of Neonatology, Beatrix Children’s Hospital, University Medical Centre Groningen, Groningen, the Netherlands; 11 Care4Neo, Neonatal Patient and Parent Advocacy Organization, Rotterdam, the Netherlands; 12 Department of Pediatric Infectious Diseases and Immunology, Wilhelmina Children’s Hospital, University Medical Centre Utrecht, Utrecht, the Netherlands; 13 Department of Biomedical Data Sciences, Leiden University Medical Centre, Leiden, the Netherlands; 14 Department of Clinical Chemistry, Erasmus University Medical Centre, Rotterdam, the Netherlands; 15 Department of Laboratory Medicine, Amsterdam University Medical Centres, Amsterdam, the Netherlands; 16 Department of Epidemiology and Data Science, Amsterdam University Medical Centres, Amsterdam, The Netherlands

**Keywords:** Preterm infants, Early-onset sepsis, Antibiotics, Biomarker

## Abstract

**Background:**

Accurate and rapid diagnosis of early-onset neonatal sepsis (EOS) in preterm infants remains problematic due to a lack of specific symptoms and diagnostic tools. Following the Dutch EOS guidelines, over 80% of infants born < 32 weeks of gestation are empirically started on antibiotics directly after birth, while the actual incidence of EOS varies between 1 and 2%. Unnecessary antibiotic exposure leads to severe short and long-term complications. The biomarker presepsin, also known as soluble CD14 subtype, may be used to reduce antibiotic prescription in preterm infants after birth. The objective of this study is to investigate whether adding a presepsin-guided decision to the guideline safely reduces antibiotic exposure directly after birth in preterm infants. Secondly, the diagnostic accuracy of presepsin for EOS will be evaluated.

**Methods:**

The PRESAFE trial is a multicentre, randomized controlled trial (RCT), including a concurrent observational study. Presepsin levels are determined from umbilical cord blood or during the first regular blood draw in all infants born < 32 weeks gestation. Infants who qualify for empirical antibiotics according to the Dutch EOS guideline are categorized as moderate or high risk for EOS based on prespecified high-risk criteria. Infants not qualifying for empirical antibiotics are categorized as low risk. Preterm infants with a moderate risk for EOS are randomized 1:1 into an intervention arm and a comparator arm. In infants allocated to the intervention arm, empirical antibiotics will only be started above a prespecified presepsin level of ≥ 645 pg/ml. In the comparator arm, infants will be treated according to the standard of care following the Dutch EOS guideline, equivalent to starting empirical antibiotics. The co-primary outcomes of the RCT are the incidence of culture-proven EOS (non-inferiority) and unnecessary antibiotics administration (superiority). The required sample size for the RCT is 900 patients. Infants with a high- or low-risk of EOS are excluded from randomization but included in a concurrent observational study and treated according to the Dutch EOS guideline. The primary outcome of this part is the diagnostic accuracy of presepsin.

**Discussion:**

The findings of the RCT will provide evidence for safe and effective reduction of administration of antibiotics for suspected EOS in infants born < 32 weeks of gestation. The observational study will provide more insight in the diagnostic accuracy of all infants born < 32 weeks of gestation.

**Trial registration:**

ClinicalTrials.gov NCT06100614. First registered on October 25, 2023.

## Administrative information

Note: the numbers in curly brackets in this protocol refer to SPIRIT checklist item numbers. The order of the items has been modified to group similar items (see http://www.equator-network.org/reporting-guidelines/spirit-2013-statement-defining-standard-protocol-items-for-clinical-trials/).

**Table:** 

Title {1}	Presepsin to safely reduce antibiotics in preterm infants (PRESAFE): study protocol for a randomized controlled trial with a concurrent observational study.
Trial registration {2a and 2b}	ClinicalTrials.gov NCT06100614. First registered on October 25, 2023. https://clinicaltrials.gov/study/NCT06100614?term=anti-bacterial%20agents%20AND%20EOS&viewType=Table&rank=6#study-overview
Protocol version {3}	Study protocol version 4.1 – November 2024
Funding {4}	The trial is funded by The Netherlands Organisation for Health Research and Development (ZonMW) with the The Good Use of Medicines Programme round 12 in 2023 (file number 10140022210043).
Author details {5a}	^1^ Department of Neonatology, Emma Children’s Hospital, Amsterdam University Medical Centres, Amsterdam, the Netherlands ^2^ Amsterdam Reproduction & Development, Amsterdam, the Netherlands ^3^ Department of Neonatal and Paediatric Intensive Care, Division of Neonatology, Erasmus University Medical Centre, Rotterdam, The Netherlands ^4^ Department of Neonatology, Wilhelmina Children’s Hospital, University Medical Centre Utrecht, Utrecht, the Netherlands ^5^ Department of Neonatology, Willem-Alexander Children’s Hospital, Leiden University Medical Centre, Leiden, the Netherlands ^6^ Department of Neonatology, MosaKids Children’s Hospital, Maastricht University Medical Centre, Maastricht, the Netherlands ^7^ Department of Neonatology, Amalia Children’s Hospital, Radboud University Medical Centre, Nijmegen, the Netherlands ^8^ Department of Neonatology, Isala Women and Children’s Hospital, Zwolle, the Netherlands ^9^ Department of Neonatology, Maxima Medical Centre, Veldhoven, the Netherlands ^10^ Department of Neonatology, Beatrix Children’s Hospital, University Medical Centre Groningen, Groningen, the Netherlands ^11^ Care4Neo, Neonatal Patient and Parent Advocacy Organization, Rotterdam, the Netherlands ^12^ Department of Pediatric Infectious Diseases and Immunology, Wilhelmina Children’s Hospital, University Medical Centre Utrecht, Utrecht, the Netherlands ^13^ Department of Biomedical Data Sciences, Leiden University Medical Centre, Leiden, the Netherlands ^14^ Department of Clinical Chemistry, Erasmus University Medical Centre, Rotterdam, the Netherlands ^15^ Department of Laboratory Medicine, Amsterdam University Medical Centres, Amsterdam, the Netherlands ^16^ Department of Epidemiology and Data Science, Amsterdam University Medical Centres, Amsterdam, The Netherlands
Name and contact information for the trial sponsor {5b}	This is an investigator-initiated trial and the sponsor is the Amsterdam University Medical Centres
Role of sponsor {5c}	N/A, see 5b.

## Introduction

### Background and rationale {6a}

Sepsis is one of the leading causes of neonatal morbidity and mortality [1]. Accurate and rapid diagnosis of early-onset neonatal sepsis (EOS), defined as sepsis onset within 72 h after birth, remains problematic mainly due to the non-specific signs and symptoms, and lack of reliable, rapid diagnostic tools. In the Netherlands, the national EOS guideline is used for the decision to start empirical antibiotics after birth. This guideline follows a risk-based approach including maternal and neonatal clinical risk factors [2]. Studies have shown that over 80% of preterm infants born below 32 weeks’ gestation are empirically started on antibiotics directly after birth, while the actual incidence of culture-proven EOS varies between 1 and 2% in this vulnerable population [3–5]. In other words, for every culture-proven EOS case, approximately 80 uninfected preterm infants are unnecessarily exposed to antibiotics directly after birth. Once started, antibiotics will be discontinued within 3 days in 70% of these infants, while in the remaining cases antibiotics are continued even in the absence of a culture-proven infection [3, 4]. Short-term complications of unnecessary antibiotic exposure in preterm infants are well described in the literature and include an increased risk for necrotising enterocolitis (NEC), late-onset sepsis (LOS), death, and antibiotic resistance [5–7]. A recently published national prospective observational study including 1259 preterm infants showed that those infants treated with antibiotics had a twofold increased risk for NEC, compared with the group of infants not treated with antibiotics [8]. Furthermore, a large Canadian observational study in preterm infants (*n *= 14,207) showed an association with a significantly increased risk for the composite outcome of severe intraventricular hemorrhage (IVH), LOS, NEC, retinopathy of prematurity (ROP), bronchopulmonary dysplasia (BPD), and death in infants exposed to antibiotics [9]. Potential long-term morbidities of early antibiotic use include a higher risk for asthma, allergy, and obesity due to aberrations in microbial colonization [10, 11].

Presepsin, also known as soluble CD14 subtype, is a novel biomarker showing rapid increase in blood after infection onset and having a high specificity for bacterial infections. Two recent meta-analyses showed a pooled sensitivity of 81–93% and specificity of 86–91% of presepsin for the diagnosis of EOS in neonates [12, 13]. Our prospective multicentre study provided a cut-off value for preterm infants that could significantly reduce antibiotic exposure without missing EOS cases, i.e. 645 pg/ml with 100% sensitivity and 54% specificity [14]. Before implementing presepsin in daily practice, a multicentre randomized controlled trial (RCT) is needed to confirm the hypothesis that adding a presepsin-guided decision to the Dutch EOS guideline is safe and effective in reducing antibiotic exposure.

For the patients assessed at low or high risk of EOS based on (lack of) risk factors and overt clinical symptoms, presepsin is preferably evaluated in an observational setting as diagnostic accuracy data in these groups are lacking. For both patient groups, these data could lead to improvement of the current Dutch EOS guideline. Currently, the sensitivity of the Dutch guideline for culture proven sepsis is approximately 50% in late preterm and term infants [15]. In high risk patients, the risk of withholding antibiotics for EOS is eliminated by means of the observational setting. This will ultimately contribute to a safe implementation of presepsin in all patients < 32 weeks of gestation.

### Objectives {7}

The objective of the randomized part of the study is to investigate whether adding a presepsin-guided decision to the Dutch EOS guideline safely reduces unnecessary empirical antibiotic exposure directly after birth in preterm infants born < 32 weeks of gestation categorized as moderate risk for EOS. Secondary, the objective of the observational study is to evaluate the diagnostic accuracy of presepsin in all preterm infants < 32 weeks of gestation.

### Trial design {8}

**Fig. 1:**
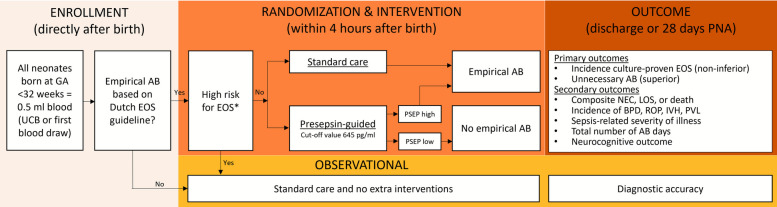
Flowchart of the study design

This is a multicentre, parallel groups, superiority and non-inferiority RCT with a concurrent observational study and is visualized in Fig. 1. The allocation ratio of the RCT is 1:1 in the intervention and comparator arm.

## Methods: participants, interventions and outcomes

### Study setting {9}

The setting of the PRESAFE study will be in all nine Neonatal Intensive Care Units (NICU) in the Netherlands, and per September 25th 2024, the first centres have started including patients. The study is performed in collaboration with the Neonatology Network Netherlands (N3) organization (https://neonatology.eu/).

### Eligibility criteria {10}

All infants born at a gestational age of < 32 weeks are eligible for enrolment. After birth, as part of standard care, all infants will be classified into low, moderate, or high risk of EOS (Table 1) [2].

**Table 1:** Classification of infants < 32 weeks in low, moderate or high risk of EOS

Low risk	Moderate risk	High risk—at least one of the criteria below
No indication to start empirical antibiotics according to the Dutch EOS guideline	Indication to start empirical antibiotics according to the Dutch EOS guideline, but no presence of high risk criteria (right column)	Suspected or proven EOS in other infants (in case of multiple births) or infants born to mothers with previous sibling with group B streptococcus disease/infection
		Suspected or confirmed diagnosis of maternal sepsis
		Unexplained respiratory insufficiency requiring invasive mechanical ventilation and FiO_2_ > 0.40 or non-invasive ventilation with FiO_2_ > 0.60 at time of randomization
		Ongoing hemodynamic instability requiring inotrope medication or more than 10 ml/kg fluid bolus at the timing of randomization
		Strong clinical concern for sepsis due to physical exam findings (i.e. minimal responsiveness, poor tone)

*AB *antibiotics, *BPD* bronchopulmonary dysplasia, *EOS *early-onset sepsis *GA *gestational age, *IVH* intraventricular hemorrhage, *LOS* late-onset sepsis, *NEC* necrotizing enterocolitis *PNA* postnatal age, *PSEP* presepsin, *PVL* periventricular leukomalacia, *ROP* retinopathy of prematurity, *UCB* umbilical cord blood

The moderate risk infants will be included in the randomized part of the study; the low and high risk infants are to be included in the concurrent observational part of the study. Exclusion criteria comprise absence (e.g. language barrier) or decline of informed consent.

### Who will take informed consent? {26a}

The study site coordinator or treating physician will inform parents and/or caregivers on the study verbally and obtain informed consent prenatally when applicable/possible. As the PRESAFE trial is a study that requires a time critical consent (randomization within 4 h after birth), we will use a two-stage consent pathway when there is no opportunity to approach women during the prenatal period. The two-stage consent pathway consists of a verbal agreement preferably before presepsin analysis and randomization, followed by written informed consent within 48 h after birth. If there is no opportunity to request verbal assent within 4 h after birth, a deferred consent procedure will be used with written consent obtained at the latest within 48 h after birth. In this situation, presepsin analysis, randomization, and possible treatment will be started before parents have been informed.

### Additional consent provisions for collection and use of participant data and biological specimens {26b}

Informed consent will also be obtained on the storage and possible future use of residual material from the collected blood.

## Interventions

### Explanation for the choice of comparators {6b}

N/A, see "Background and rationale {6a}".

### Intervention description {11a}

Infants at moderate risk of EOS will be randomly assigned (1:1 ratio) to the intervention or comparator arm. In the intervention arm, empirical antibiotics will only be started when the presepsin level is ≥ 645 pg/ml [14]. The result of the presepsin test will be communicated with the attending physicians as positive or negative without mentioning the exact level. Infants allocated to the comparator arm and those who will not be randomized will be treated according to standard care following the Dutch EOS guideline, with the treating physician and research team blinded to the presepsin test result.

### Criteria for discontinuing or modifying allocated interventions {11b}

In case of clinical deterioration of infants not receiving antibiotics allocated in the RCT intervention arm (based on a presepsin level under the threshold), the treating physician can decide to start antibiotic treatment after performing a sepsis evaluation (blood culture) if the infant fulfills at least one of the high risk criteria (Table 1). A second blood culture does not have to be performed when antibiotic treatment is started within 12 h after birth and thus after the first blood culture.

### Strategies to improve adherence to interventions {11c}

N/A, see "Criteria for discontinuing or modifying allocated interventions {11b}".

### Relevant concomitant care permitted or prohibited during the trial {11d}

There is no relevant specific care permitted or prohibited during the trial.

### Provisions for post-trial care {30}

N/A, since there is no specific post-trial care other than regular follow-up at outpatient clinic.

### Outcomes {12}

#### Primary outcomes

The co-primary outcomes of the RCT part of the study are (1) the incidence of culture-proven EOS (non-inferiority) and (2) unnecessary antibiotics prescription started within the first 72 h after birth, i.e. ≤ 3 days of antibiotic administration (superiority). Antibiotic administration after birth is started to pre-emptively treat EOS. By adding a presepsin-guided decision to the Dutch EOS guideline for those infants qualifying for antibiotic treatment, the rate of antibiotic administration can presumably be reduced. However, it is imperative that this reduction in antibiotics in the RCT part of the study may not be outweighed by an increase in untreated (culture proven) EOS.

For the diagnostic accuracy of presepsin, we will include infants with moderate and high risk for EOS, directly after birth. The target condition is culture-proven sepsis. Accuracy measures that will be estimated are sensitivity, specificity, positive predictive value, negative predictive value, and area under the curve. The infants in the low-risk group will only be used for exploring the specificity of presepsin, as no standard blood culture will be drawn in this group.

#### Secondary outcomes

Secondary outcomes will only be evaluated in the RCT part of the study and will be collected from birth until discharge home. Secondary outcomes include the following:

- Sepsis-related severity of illness using the neonatal Sequential Organ Failure Assessment (nSOFA) score in infants treated with antibiotics > 3 days [16]. Severe illness is defined as a nSOFA score of ≥ 4 within the first 3 days of antibiotic treatment. The nSOFA score will be calculated 3 times a day within the first 3 days of antibiotic treatment. A cut-off value of 4 will be used to differentiate between severe illness and non-severe illness.
- Incidence of meningitis and sepsis-related mortality [16, 17]
- Total number of antibiotic days when started < 72 h after birth.
- Incidence of composite outcome NEC stage II or III, LOS or death, and its separate components [18]
- Incidence of moderate and severe BPD, including postnatal steroid prescription for BPD and total ventilation days [19, 20]
- Incidence of intraventricular hemorrhage (IVH) including posthemorrhagic ventricular dilatation (PHVD) and/or periventricular leukomalacia (PVL) [21]
- Incidence of retinopathy of prematurity (ROP) [22]

Neurodevelopmental outcome will be assessed in the included infants qualifying for regular long-term follow-up program at 2, 5.5, and 8 years corrected age, i.e. all infants with GA < 30 weeks, birth weight < 1000 g, or birth weight < 1500 g classified as small for GA. Neurodevelopmental impairment is defined as follows: a cognitive and/or motor composite score less than 85 on the BSID-IV-NL; cerebral palsy greater than level II on the Gross Motor Function Classification System; hearing or visual impairment [23]. Lastly, an economic evaluation will be performed alongside the clinical study to estimate cost-effectiveness (defined as costs per unnecessary antibiotic prescription). The costs and benefits of an added presepsin-guided decision to the Dutch EOS guideline will be compared with care as usual (e.g. including medical costs of hospital days, antibiotic use, laboratory tests and labour costs).

### Participant timeline {13}

**Table:** 

Time point	Study period								Follow-up
	Prenatal postnatal allocation post-allocation discharge								
			0–4 h after delivery	0 h	48 h	72 h	168 h	> 28 days PNA	2, 5.5, 8 years
**Enrollment**									
Eligibility screen	X	X							
Informed consent	X	X		X	X				
Allocation			X						
**Standard care**									
Empiric antibiotics Blood culture Presepsin analysis		X	X	X	X	X	X		
**Intervention (presepsin-guided)**									
Empiric antibiotics				X	X	X	X		
No antibiotics				X	X	X	X		
Escape antibiotics Blood culture Presepsin analysis		X	X	X	X	X	X		
**Assessments**									
Blood sample*			X						
Sepsis-related severity of illness				X	X	X	X		
Culture-proven EOS				X	X	X			
Incidence of NEC, LOS, death								X	
Incidence BPD, ROP, IVH, PVL								X	
Demographics, clinical variables	X	X	X	X	X	X	X		
Neurodevelopmental outcome								X	X
Economic evaluation				X	X	X	X	X	X

SPIRIT figure. This participant timeline applies for patients in the RCT part of the study

*0.5 mL umbilical cord blood or during first regular blood draw

*BPD* bronchopulmonary dysplasia, *EOS* early-onset sepsis, *IVH* intraventricular haemorrhage, *LOS* late-onset sepsis, *NEC* necrotizing enterocolitis, *PNA* postnatal age, *PVL* periventricular leukomalacia, *ROP* retinopathy of prematurity

### Sample size {14}

Incidence of culture-proven EOS is assumed to be 1.4% based on the most recent and largest cohort with identical GA (< 32 weeks, *N* = 43,178) [4] and confirmed by screening 4 years admission data (2016–2019) of the NICU of Erasmus MC (Rotterdam, The Netherlands). With a plasma presepsin cutoff of 645 pg/ml, sensitivity is 100% (95% CI 78–100%) and specificity is 54% (95% CI 41–62%) for detecting EOS [14]. To balance the feasibility and the safety of the study, we have chosen a non-inferiority margin of 2% for the absolute between-RCT-arms difference in culture-proven EOS incidence (intervention–control) [24]. We deem this extra absolute risk acceptable in light of the incremental daily increased risk of adverse events with administration of antibiotics (NEC, LOS, death) [7]. Assuming there is no difference in culture-proven EOS between both study groups, a sample size of 427 is needed in both study groups (total *N* = 854), using a two-group, large-sample normal approximation test of proportions with 80% power and 1-sided 5% significance level. For the co-primary superiority outcome unnecessary antibiotics prescription < 72 h after birth, it is estimated that approximately 70% of all infants in the control group will discontinue antibiotics within 72 h (ref). Based on expert opinion, we consider an absolute reduction of 30% or more clinically relevant. Based on the aforementioned diagnostic accuracy of a presepsin cut-off level of 645 pg/ml, with a number of 427 in both study groups, we expect to detect a 30% reduction in the co-primary superiority outcome with a power of 99% and a 2-sided alpha of 0.05 (two-group Fisher’s-exact test). To allow for some unassessable cases (e.g. based on technical issues or withdrawn consent, estimated at 5%), a number of 450 will be included in both RCT-arms. The total study population is estimated to consist of 20% low risk (*N* = 244), 70% moderate risk (*N* = 900), and 10% high risk infants (*n* = 122).

### Recruitment {15}

N/A, as all infants < 32 weeks admitted to the NICU are eligible for inclusion.

## Assignment of interventions: allocation

### Sequence generation {16a}

Randomization will be performed centrally, using web-based block randomization stratified by study site and GA with random permuted blocks within the strata (random block size varying between 4 and 8) with a 1:1 allocation ratio. Randomization sequence is generated using Castor EDC (2019, Castor Electronic Data Capture [online], available at https://castoredc.com), which is regulatory compliant software, ensuring allocation concealment. Multiples (i.e. siblings) will be randomized to the same treatment arm if they both qualify for randomization.

### Concealment mechanism {16b}

N/A, see "Sequence generation {16a}".

### Implementation {16c}

N/A, see "Sequence generation {16a}".

## Assignment of interventions: blinding

### Who will be blinded {17a}

N/A, allocation is not blinded in this study.

### Procedure for unblinding if needed {17b}

N/A, allocation is not blinded in this study.

## Data collection and management

A Trial Master File will be made before the start of the study, with all the essential documents in accordance with Good Clinical Practice guidelines. All data and body-material will be handled confidentially and pseudonymized. A unique subject identification code will be generated for each participant and is not based on the name and day of birth. Body-material will be coded with a barcode before storage in the freezer. The principal investigator of each site will safeguard the key to the unique subject identification code. The key will be stored on a secured drive at the local hospital and is only accessible for the local principal investigator, monitor, auditor, and Health and Youth Care Inspectorate. Study data will be stored in a Castor EDC database, which is regulatory compliant software, handled according to the Personal Data Protection Act. Clinical data will be stored for 15 years and body-material for 5 years. All of the above is registered in a detailed data management plan according to the FAIR principles.

### Plans for assessment and collection of outcomes {18a}

See main heading “Data collection and management”.

### Plans to promote participant retention and complete follow-up {18b}

N/A, patient retention is secured through hospital admission, and long-term follow-up data are dependent on visits at the outpatient clinic as part of standard care.

### Data management {19}

See main heading “Data collection and management”.

### Confidentiality {27}

See main heading “Data collection and management”.

### Plans for collection, laboratory evaluation and storage of biological specimens for genetic or molecular analysis in this trial/future use {33}

See main heading “Data collection and management”.

## Statistical methods

### Statistical methods for primary and secondary outcomes {20a}

Analyses for both co-primary outcomes will be performed using the intention-to-treat principle and additionally using the per-protocol principle for the co-primary non-inferiority outcome to evaluate consistency of both analysis approaches compliant with recommendations for non-inferiority analyses [25]. The absolute between-RCT-groups difference in the co-primary non-inferiority outcome (incidence of culture-proven EOS) (intervention—control) with its one-sided 95% confidence interval (CI) will be calculated. When the upper limit of this one-sided 95% CI is below 2%, non-inferiority can be claimed. The between-RCT-groups difference in proportions of the co-primary superiority outcome (unnecessary antibiotics administration) will be studied under a common superiority hypothesis and expressed as a 2-sided 95% CI and analyzed using Fisher’s exact test and mixed effects modeling with site as a random effect and GA as fixed factor (2-sided *p* < 0.05 considered statistically significant). Analyses of secondary outcomes will be performed according to the intention-to-treat principle using common two-tailed hypothesis tests with alpha equal to 0.05 and using two-sided 95% CIs. Because the analyses of the secondary outcomes are considered exploratory, no correction for type I error will be made. Differences between groups in continuous variables will be analyzed with Student’s *t*-test or, if continuous data is not normally distributed, the Mann–Whitney *U* test will be used. Categorical variables will be compared with the chi-squared test or Fisher’s exact test, as appropriate. Furthermore, mixed effects modeling will be performed with site as a random effect and including randomization stratification factors as appropriate.

The observational part of the study will focus on diagnostic accuracy analysis of presepsin, such as sensitivity, specificity, positive predictive value, negative predictive value, and receiver operating characteristic curves with optimal cut-off values per subgroup.

A detailed statistical analysis plan comprising all study outcomes will be completed before database lock and published separately.

### Interim analyses {21b}

An interim analysis is planned when half (50%) of the included patients have completed follow-up up till discharge home and will be performed by an independent statistician of the Data Monitoring Committee (DMC). Additional reviews will be conducted at the DMCs request. The DMC will make their final recommendations regarding the results of interim data analyses and may recommend early trial termination based on unanticipated safety concerns. The justifications for a recommendation to terminate the study prematurely due to clear harm will be based on data showing a notably increased (serious) adverse events in the intervention group. The primary focus of the interim analysis is a safety analysis evaluating the overall mortality during hospital admission in both RCT groups. The other safety outcomes are supportive safety data.

Halfway the study (i.e. when the first 450 included participants have completed follow up for their hospital admission), recommendations for stopping for overall mortality will be based on the following pre-specified formal stopping rule. The overall mortality rate in infants born below 32 weeks GA is estimated ~ 10% (based on data of the years 2019–2022 of the national registration program “Perined”). Assuming an incidence of mortality of 10% in the control group, if the incidence of mortality in the intervention group is at least 50% higher relatively (i.e. > 15%, relative risk > 1.5), this will be considered a warning sign for a safety concern and a justification for recommendation to stop the study prematurely. If this situation occurs, further inspection of the results will be incorporated in the decision whether or not to stop the study prematurely. This includes inspection of the intervention group to determine whether the excess mortality can be attributed to the presepsin-low group (not started with antibiotics) and/or the presepsin-high group (started with antibiotics). Also the relation of the other safety outcomes with the study intervention, and the overall study conduct will be taken into account by the DSMB to decide whether the trial can be continued unchanged, needs closer monitoring, or should be stopped.

The DSMB may also recommend cessation of the trial if it is unable to successfully recruit participants, encounters improper data handling, or is not able to successfully implement study protocols. A formal interim analysis for efficacy (inferential testing) will not be conducted.

### Methods for additional analyses (e.g. subgroup analyses) {20b}

Subgroup analyses are planned for GA 24–28 weeks versus 28–32 weeks and SGA patients. Subgroup analyses and any other additional analyses will be further specified in the statistical analysis plan, which will be completed before database lock and published separately. A prospective economic evaluation will be performed in line with the randomized part of the study, providing insight into the value of the addition of a presepsin-guided step to the Dutch EOS guideline by reducing unnecessary antibiotic exposure directly after birth in preterm infants born < 32 weeks GA and at moderate risk of EOS. The executed cost-effectiveness analysis will reflect the health care costs and direct and indirect consequences expressing the benefits of the presepsin-guided step based on current practice (Dutch EOS guideline) compared to the new strategy.

### Methods in analysis to handle protocol non-adherence and any statistical methods to handle missing data {20c}

Analyses will be performed using the intention-to-treat principle and also using the per-protocol principle for the co-primary non-inferiority outcome to evaluate the consistency of both analysis approaches compliant with recommendations for non-inferiority analyses [25]. The per-protocol population will exclude patients with major protocol deviations, which may have an impact on the evaluation of the co-primary non-inferiority outcome; these will be defined in the statistical analysis plan before the database lock.

No missing data for the primary outcome are expected and therefore methods such as imputation are not anticipated.

### Plans to give access to the full protocol, participant level-data and statistical code {31c}

Metadata will be published on Amsterdam UMC DataverseNL after publication at the end of this research project. All data generated during the study, including participant-level data and statistical code, are available upon reasonable request with the corresponding author.

## Oversight and monitoring

### Composition of the coordinating centre and trial steering committee {5d}

The trial steering committee (TSC) includes the principal investigator of each participating centre, two additional investigators from the coordinating centre, and N3 representatives. The TSC has scientific responsibility for the study and is the main decision-making body of the study. Furthermore, overall supervision of the study will be provided by the TSC.

### Composition of the data monitoring committee, its role and reporting structure {21a}

An independent DMC, consisting of a statistician, pediatric infectious disease specialist, neonatologist, and representative of the patient and parent organization Care4Neo, forms the DMC. The DMC will have access to unblinded study data to allow for interim data analyses. The DMC will make their final recommendations regarding the results of interim data analysis and may recommend early trial termination with unanticipated safety concerns (see paragraph "Interim analyses {21b}"). They may also recommend cessation of the trial if it is unable to successfully recruit participants, encounters improper data handling, or is not able to successfully implement study protocols.

The advice(s) of the DSMB will only be sent to the principal investigator of the study. Should the principal investigator decide not to fully implement the advice of the DSMB, the principal investigator will send the advice to the reviewing MERC, including a note to substantiate why (part of) the advice of the DSMB will not be followed.

### Adverse event reporting and harms {22}

Serious adverse events (SAEs) will be reported through the web portal *Onderzoeksportaal* from the Central Committee on Research Involving Human Subjects (CCMO) to the accredited MREC within 7 days of first knowledge. These SAEs are defined as sepsis-related mortality and start of antibiotic therapy in patients that were initially not started on antibiotics in the presepsin-guided therapy intervention arm. This study population (critically ill preterm infants) has a high risk of other serious complications (so-called “context-specific SAE’s”), which are inherent to their vulnerable condition and unrelated to the intervention that is under evaluation in this trial. These complications are included in the primary and secondary outcomes of this study and are recorded in the Case Report Form. This documentation will include the date of diagnosis, classification/gradation of the complication, type of action taken if appropriate (with some complications a wait and see approach is warranted). In light of the above, immediate and individual reporting of all these condition-related complications will not enhance the safety of the study. Once a year, an overview of the aforementioned complications for each treatment arm and ordered by organ system will be presented to the DMC and MREC.

### Frequency and plans for auditing trial conduct {23}

The study will be monitored by clinical research associates employed by certified monitor organizations and by means of personal visits to investigator’s facilities and other communications (e.g. telephone calls and written correspondence). The visits will be conducted depending on a prespecified number of included study subjects and comprises review of informed consent procedures, source data review and verification, study protocol adherence, adherence to GCP and applicable national regulations. All monitoring agreements and activities are specified in a separate monitoring plan.

### Plans for communicating important protocol amendments to relevant parties (e.g. trial participants, ethical committees) {25}

All substantial amendments to the study protocol will be notified to the MREC and local principal investigators. Other non-substantial amendments will not be notified to these parties but will be recorded and filed by the coordinating centre.

## Dissemination plans {31a}

The results of the study will be published in peer-reviewed journals, the website of the Dutch Society of Pediatrics, and websites of collaborators such as N3 (Neonatal Network Netherlands) and the parent association Care4Neo. Additionally, the results will be presented at international conferences and will be incorporated in national and international early-onset sepsis guidelines.

## Discussion

The PRESAFE study focusses on a biomarker-driven strategy to safely reduce unnecessary antibiotic exposure in uninfected preterm infants by means of both a randomized and observational study. Nowadays, antibiotic stewardship efforts focus primarily on shortening the duration of antibiotic prescription and do not address the possibility of not starting antibiotics in preterm infants [26, 27]. This has recently changed with the NICU Antibiotics and Outcomes (NANO) trial, a multicentre double blinded RCT in the United States of America and Canada [6]. This trial is currently recruiting 802 preterm infants (GA < 31 weeks) to evaluate the effect of reducing empirical antibiotic use at birth on adverse outcomes of prematurity in preterm infants at modest risk of EOS. Participants in this RCT are randomized to receive antibiotics or placebo. Low and high EOS risk patients are excluded from this trial, similarly to our approach. The investigators of the NANO trial hypothesize that the benefits of treating all patients at moderate risk of a perinatal infection do not outweigh the adverse effects (LOS, NEC, or death) of unnecessary empirical antibiotics after birth in this specific age group [6]. Although we underline the hypothesis of an increased risk of adverse outcomes during initial hospitalization after unnecessary treatment with antibiotics, not treating this vulnerable population with moderate risk for EOS might increase the risk of missing an EOS with potential severe consequences for the individual patient. Therefore, we hypothesize that adding an early and accurate biomarker for those infants with moderate risk of a perinatal infection may be beneficial in safely reducing unnecessary antibiotic exposure in all preterm infants born < 32 weeks gestational age (GA) without posing additional danger of untreated EOS.

The diagnostic value of other biomarkers used in daily care like C-reactive protein (CRP), procalcitonin (PCT), and different interleukins has been studied for this purpose, but all lack sufficient accuracy at initial EOS suspicion [28]. In contrast, increasing evidence is showing that presepsin (soluble CD14 subtype) is a very promising biomarker for this purpose [12, 13]. Presepsin is immediately released from the macrophage after binding with the bacteria, which makes this biomarker hypothetically very specific for bacterial infections, and concentrations increase rapidly. Presepsin levels can be measured by a rapid chemiluminescent enzyme immunoassay (Mitsubishi Chemical Medicine Corporation, Tokyo, Japan) using plasma, with results available within minutes. A recent Dutch multicentre observational diagnostic accuracy study was performed to determine the optimal cut-off value of presepsin to detect EOS in preterm infants with an indication for empiric antibiotic therapy (*n* = 169). A sensitivity of 100% (95% confidence interval (CI) 78–100%) was reached with a specificity of 54% (95% CI 41–62%) at a cut-off value of 645 pg/ml (area under the curve (AUC) 0.84; 95% CI 0.73–0.95) [14].

In the study design phase, we envisioned several potential challenges for the PRESAFE trial and concurrent observational study. The emergency context of preterm birth forms a hazard for the recruitment and informed consent process. With the help of our MREC and Care4Neo as patient and parent representation, we sketched and explored different scenarios, resulting in a set of different options to acquire informed consent, based on what is most suitable per patient. Another important challenge for the PRESAFE trial is in the patient classification according to the Dutch EOS guideline and classification of high-risk criteria. In our experience, there is a considerable variation in guideline interpretation amongst different clinicians and centres. Mandatory classification before randomization in eCRF supports minimization of selection bias. Furthermore, as the incidence of culture-proven sepsis is low, the experience of safely not giving empirical antibiotics in the presepsin-guided therapy arm of the randomized study may lead to a tendency to classify patients as low risk for EOS.

## Trial status

MREC Study protocol version number is 4.1, dated November 2024. Recruitment began 25–9–2024 and is expected to be completed late 2027.

## Data Availability

The final trial dataset will be retained and archived for a minimum of 15 years after study completion. All data acquired in the study is available from the corresponding author at reasonable request.

## Abbreviations

BPD: Bronchopulmonary dysplasia
CRP: C-reactive protein
DMC: Data Monitoring Committee
EOS: Early-onset sepsis
GA: Gestational age
IVH: Intraventricular hemorrhage
LOS: Late-onset sepsis
MREC: Medic Research Ethics Committee
NEC: Necrotizing enterocolitis
PCT: Procalcitonin
PVL: Periventricular leukomalacia
RCT: Randomized controlled trial
ROP: Retinopathy of prematurity
SAE: Serious adverse event
SGA: Small for gestational age
TSC: Trial steering committee
